# Laser-Treated Surfaces for VADs: From Inert Titanium to Potential Biofunctional Materials

**DOI:** 10.34133/2022/9782562

**Published:** 2022-07-13

**Authors:** Eduardo Bock, Wilhelm Pfleging, Dayane Tada, Erenilda Macedo, Nathalia Premazzi, Rosa Sá, Juliana Solheid, Heino Besser, Aron Andrade

**Affiliations:** ^1^Laboratory of Bioengineering and Biomaterials, Federal Institute of Technology in Sao Paulo (IFSP), Sao PauloBrazil; ^2^Center of Engineering in Circulatory Assistance, Institute Dante Pazzanese of Cardiology (IDPC), Sao Paulo, Brazil; ^3^Institute for Applied Materials-Applied Materials Physics, Karlsruhe Institute of Technology (KIT), Karlsruhe, Germany; ^4^Federal University of Sao Paulo (UNIFESP), Sao Jose dos Campos, Brazil; ^5^National Institute for Space Research (INPE), Sao Jose dos Campos, Brazil

## Abstract

*Objective*. Laser-treated surfaces for ventricular assist devices. *Impact Statement*. This work has scientific impact since it proposes a biofunctional surface created with laser processing in bioinert titanium. *Introduction*. Cardiovascular diseases are the world’s leading cause of death. An especially debilitating heart disease is congestive heart failure. Among the possible therapies, heart transplantation and mechanical circulatory assistance are the main treatments for its severe form at a more advanced stage. The development of biomaterials for ventricular assist devices is still being carried out. Although polished titanium is currently employed in several devices, its performance could be improved by enhancing the bioactivity of its surface. *Methods*. Aiming to improve the titanium without using coatings that can be detached, this work presents the formation of laser-induced periodic surface structures with a topology suitable for cell adhesion and neointimal tissue formation. The surface was modified by femtosecond laser ablation and cell adhesion was evaluated *in vitro* by using fibroblast cells. *Results*. The results indicate the formation of the desired topology, since the cells showed the appropriate adhesion compared to the control group. Scanning electron microscopy showed several positive characteristics in the cells shape and their surface distribution. The *in vitro* results obtained with different topologies point that the proposed LIPSS would provide enhanced cell adhesion and proliferation. *Conclusion*. The laser processes studied can create new interactions in biomaterials already known and improve the performance of biomaterials for use in ventricular assist devices.

## 1. Introduction

A patient who suffers from congestive heart failure may not have adequate blood circulation in organs and tissues, providing the necessary physiological supply of oxygen and nutrients. In this case, possible treatments are heart transplantation or the alternative therapy and circulatory assistance, when a blood pump is implemented as ventricular assist device (VAD) [1, 2]. Two main unwanted and inherent processes to VAD implantation are the formation of thrombus inside the pump and hemolysis. The first is due to high interaction between blood and pump’s titanium surface. The second is due to mechanical trauma that can break the cytoplasmic membrane of the red blood cells [3]. In order to avoid the thrombus generation and even minimize hemolysis, it is necessary to create a bioactive interface between titanium and blood. If the endothelial cells form a neointimal tissue, the process is considered an endothelization [4]. Titanium is the biomaterial employed to produce those pumps, and it can be enhanced to obtain an endothelial tissue growth becoming not just bioinert but biofunctional material [5, 6].

## 2. VAD Biocompatibility

The biocompatibility of a material cannot be defined by a single test, but it is bounded with the biomaterial application and its functionality [7]. During decades of VAD design, several materials have been tested. From flexible elastomers in the first pulsatile artificial hearts to materials of biological origin, the design requirements of these pumps have always been focused on materials with specific properties and their role in the equipment general functioning. Over the years, there was a paradigm shift in terms of total organ replacement, and then, in terms of pulsatility need and new ways of accelerating, the blood flows inside the pump for a physiological flow. Total artificial hearts were giving space to new technologies like VADs and pulsatile flow to continuous flow from axial and centrifugal pumps. The area of artificial organs and artificial hearts, more specifically, VAD development, is characterized by constant evolution of technologies and scientific discoveries [7, 8]. The differentiation of materials must take into account aspects of its application, whether it is in contact with blood, whether it is composed of moving parts, and whether there are friction and wear. An appropriated design should consider suitable materials in order to achieve an outstanding performance. The INTERMACS report has been showing this relationship between materials and the success of VADs, since VADs with noncontact surfaces are showing better survival rates in Kaplan-Meyer curves [2].

However, a material that is present in the vast majority of nonpulsatile VADs is titanium and its commercial alloys. From the biomaterial development point of view, titanium is considered to be inert. It means that titanium does not bring a severe response, good or bad to the host organism environment. It meets the needs for VADs since it is not a toxic material and it has appropriate mechanical properties with only few disadvantages such as weight and scale production. However, a biomaterial must be better than just inert and must promote a positive active interaction with the organism system to be considered biocompatible [7, 8].

This quest for an enhancement of the titanium performance led to several research projects that are aimed at developing biomaterials with a less leaning response to thrombus formation and hemolysis. Several types of coatings have been tested like HeartMate I with polyurethane (PU), Evaheart with methacryloyloxyethyl phosphorylcholine- (MPC-) polymer, Carmeda with heparin coating, and VentrAssist with diamond like carbon (DLC). While passive coatings serve as a barrier between the bulk material and blood, bioactive coatings directly interact with the blood or interfere with the process of intimal proliferation. One of the possibilities for improving the interaction of VAD with blood, a new disruptive technology, may be the use of biomaterials that interact positively with blood and promote the growth of epithelial tissue similar to that found inside blood vessels. Thus, considering the blood interaction, the same biological surface would be found inside the ventricle, the pump, and the aorta [8–12].

## 3. Materials and Methods

The IFSP Bioengineering and Biomaterials Laboratory developed two centrifugal blood pumps designed for ventricular assistance in partnership with the IDPC Center of Engineering in Circulatory Assistance [13–19]. In experiments with superficial modification of titanium and its alloys with Plasma Electrolytic Oxidation (PEO), positive preliminary results were obtained in the growth of epithelial tissue [4]. Alternatively, the method for producing laser-induced periodic surface structures (LIPSS) is a promising technical approach to obtain a morphology suitable for the desired tissue growth. This work seeks to report the first results of fibroblast assays with Ti after introducing of LIPSS along the surface [20–24]. Fibroblasts are the main cells involved in healing, and their main function is to maintain the integrity of the connective tissue by synthesizing the components of the extracellular matrix.

### 3.1. LIPSS

The formation of classical LIPSS was discovered in the early sixties. LIPSS are formed during laser beam exposure of materials by applying low laser fluences close to the ablation threshold. Simply spoken, LIPSS are generated by an interference effect of the incoming laser light with a laser-induced surface wave. Classical or Low Special Frequency LIPSS (LSFL) consists of periodic lines with a structure pitch distance of about the laser wavelength. The orientation of the line pattern is perpendicular to the polarization direction of the applied laser radiation. Besides LSFL, there were also so-called High Special Frequency LIPSS (HSFL) detected in the nineties by using femtosecond laser radiation instead of classical long pulsed laser systems, i.e., nanosecond or millisecond lasers. The periodicity of those HSFL can be significantly below the wavelength of the applied laser wavelength. The excitation of surface plasmon polaritons was identified as the main mechanism for formation of HSFL and LSFL. LIPSS is generated in ambient air by scanning the laser beam along the material surface leading to the creation of nano- and submicron structures which are capable to functionalize all types of materials [20, 21]. Beyond the increase of the cell growth, the LIPSS application on titanium can reduce the friction inside of the pump: directing the structures created for the flow direction, reducing the shear stress on the pump walls, and reducing the hemolysis induced by mechanical trauma [19]. Another LIPSS advantage is that it is a clean and highly controlled process, an important feature for manufacturing processes of medical devices.

**Figure 1:**
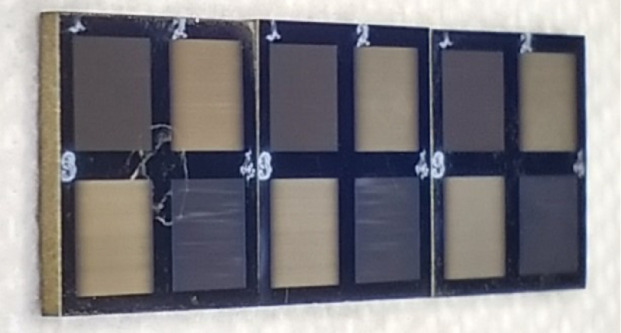
Quadrants of the samples; each quadrant has a topology obtained from different parameters showing wavelength dependent light diffraction resulting in different colors.

In order to compare four different parameters of the LIPSS manufacturing process, four quadrants were created in each sample with a femtosecond laser (Tangerine, Amplitude Systèmes, France) built in a micromachining system PS450-TO (Optec s.a., Belgium). Like a cross, separating the four quadrants, the substrate material can serve as a basis for comparison between the polished and the textured titanium for tests with fibroblasts (Figure 1) [25–29].

**Figure 2:**
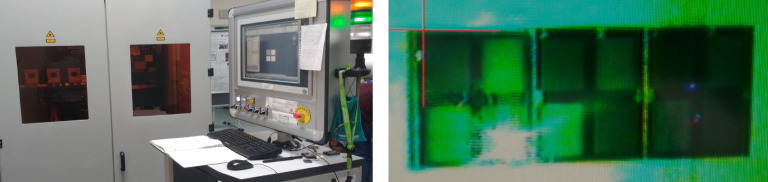
Overview about the used micromachining workstation and titanium sample which is just treated by the green laser beam (wavelength: 515 nm).

The titanium surface modification was realized using an ultrafast fiber laser (Figure 2).

LIPSS were created by using a pulse length of 400 fs, a laser wavelength of 515 nm, and f-theta lens with a focal length of 163 mm. The laser focus diameter was in the order of 20 *μ*m. Laser repetition rates up to 2 MHz with tunable pulse durations from 380 fs up to 10 ps can be processed in this equipment. The samples were modified with LIPSS formed by processing close to the energy ablation threshold of titanium with an average power of 80–120 mW, a hatch distance of 10 *μ*m, and a scanning speed of 10–50 mm/s, according to Table 1.

**Table 1:** Sample quadrants and respective LIPSS process parameters and characteristics.

	1^st^ quadrant	2^nd^ quadrant	3^rd^ quadrant	4^th^ quadrant
Laser power (mW)	120	120	120	80
Speed (mm/s)	10	50	50	10+50
Pitch (*μ*m)	15	8	12	8
Number of laser scans	1	1	2	1

**Figure 3:**
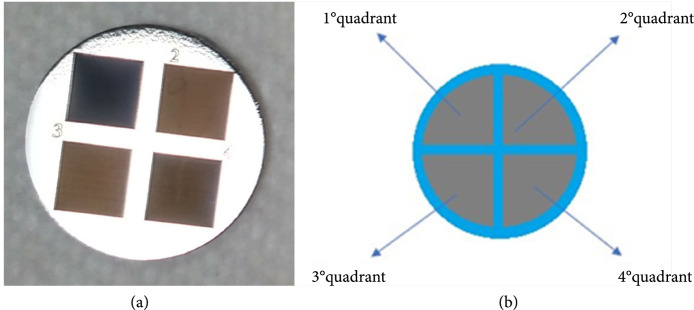
Cylindrical samples (a) and the same four quadrants topology/parameters scheme (b).

A second group of cylindrical samples with 12 mm in diameter were machined according to the scheme and photos in Figure 3 in order to better fit the 24-well cell plate for *in vitro* assays.

### 3.2. Cell Adhesion Experiments

Mouse embryonic fibroblast cells (MEF) were grown on samples of titanium in order to assess the interaction between cells and the surfaces with different topologies. Notably, the complete biocompatibility assessment would include the evaluation of the interaction with blood cells (mainly erythrocytes and red blood cells), cell proliferation assays, cytotoxicity, and genotoxicity assays. Nevertheless, in this work, the focus of the in vitro assays was on the evaluation of cytocompatibility, and therefore, it was performed with fibroblast cells. It has been pointed out the crucial role of evaluating the biological interactions and possible toxic effects on healthy cells with high differentiation capacity as fibroblasts in the development of a new biomaterial. Herein, the fibroblast cell adhesion on LIPSS titanium surface was performed in order to evaluate which pattern could be more appropriated to reach the best cell interaction.

Before the in vitro assays, the samples were sterilized by washing with ethanol 70% followed by UV irradiation (20 min) on both sides. MEF cells were grown in minimum essential medium (MEM) supplemented with 2 mM L-glutamine, 1 mM sodium pyruvate, 0.1 mM nonessential amino acids, 100 units/mL penicillin, 100 *μ*g/mL streptomycin, and 10 vol.% fetal bovine serum. After reaching 80% of confluence, the cells were seeded on the samples placed in 12-well plates. Each sample contained four quadrant samples consisting of both laser-functionalized titanium samples and control group (cross between quadrants). The plate was placed in the incubator (37°C, 5% CO_2_), and the cells were cultivated for 24 h. Following, the culture medium was withdrawn from each well, and 2.5% glutaraldehyde was added for 1 h. Then, the glutaraldehyde was removed, and successive washing steps were performed with ethanol at 10, 25, 50, 75, and 90% for 20 min each. Finally, the samples were immersed in ethanol 99% for 1 h, and after removing the samples from ethanol, they were incubated for 20 h (37°C, 5% CO_2_). The samples were sputter coated with a gold/platinum in a sputtering system (Quorum, Q150R ES) and analyzed by SEM (Fei, INSPECT S50, with Everhart-Thornley detector) available at NAPCEM-ICT UNIFESP. The control group had, except for the region located for seeding cells, the same treatment and operation performed for the others described.

## 4. Results

**Figure 4:**
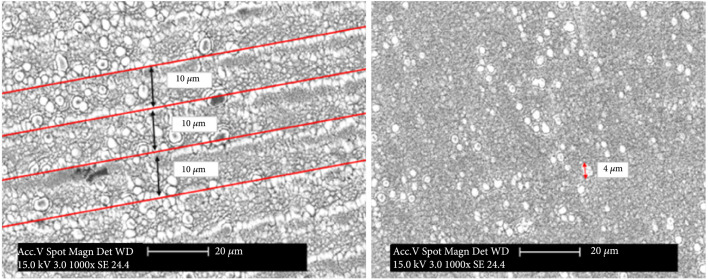
SEM (1000x magnification) of laser processed titanium showing the applied laser hatch distance (10 *μ*m) and a 4 *μ*m sized particle formed on the surface.

The laser process was able to create the desired LIPSS morphology with the chosen laser power, speed, and pitch parameters. Scanning Electron Microscopy (SEM) characterization of the surface and the topology of any quadrant in different processes described in Table 1 can be seen on Figure 4.

The applied pitch of 10 *μ*m, followed by scattered titanium oxide present on the porous surface with 4 *μ*m particles, can be observed in Figure 4. The created LIPSS have a periodicity of 500 nm (not shown here).

**Figure 5:**
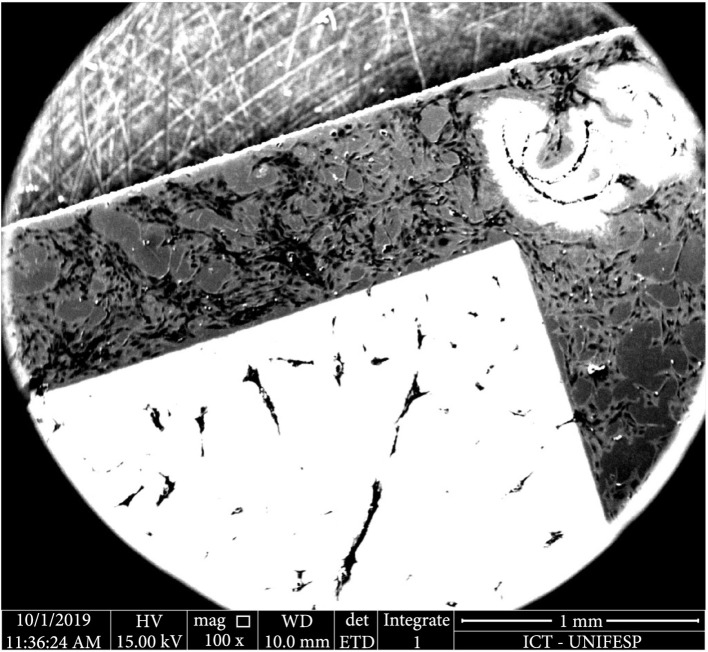
SEM (100x magnification) of the control group positioned between quadrants, mainly polished titanium.

After the incubation with MEF cells, the samples were fixed and characterized by SEM again. The cross-shaped area between the quadrants showed the results obtained with polished titanium, which was considered the control group (Figure 5).

**Figure 6:**
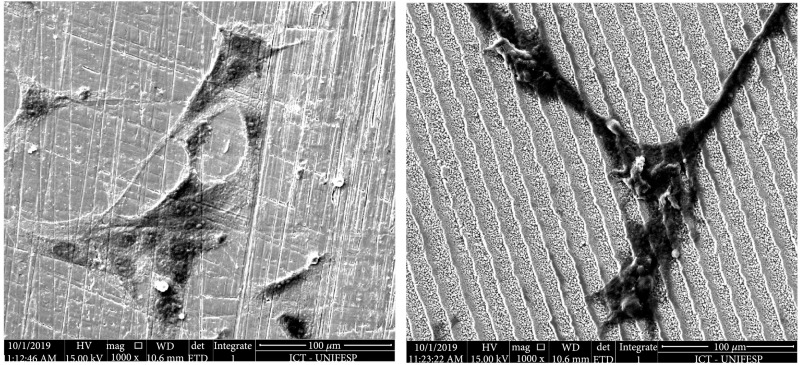
SEM of the polished titanium (left) and the created LIPSS (right) with fibroblasts grown on the same well-plate assay (both with 1000x magnification).

It is important to note that since the cylindrical samples contained 4 different quadrants separated by a polished titanium surface, the same cell suspension was simultaneously in contact with all the types of surface. This setup allowed a better comparison between the different topologies and the control group. Although the control group shows higher cell density, suggesting higher cell proliferation (Figure 5), the observation of cells at higher magnification shows similar cell morphology (Figure 6). Since the cells presented an elongated shape, which is characteristics of fibroblasts, it was possible to consider that the surface was suitable for the MEF cell adhesion and did not induce cytotoxic effects which would result in round-shaped cells.

**Figure 7:**
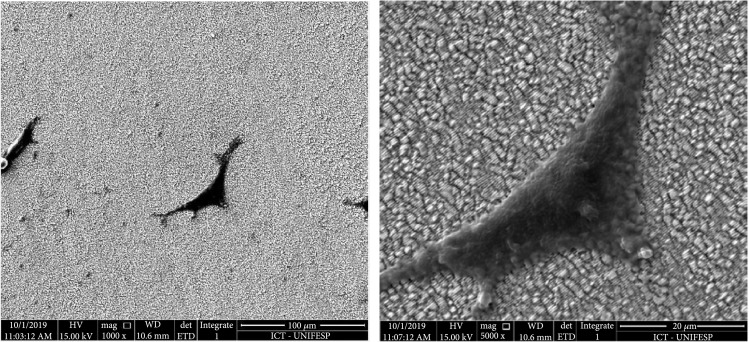
SEM of the LIPSS and fibroblasts in 1000x magnification (left) and 5000x magnification (right).

In the SEM images, it was possible to see that cell adhesion was also promoted on the other topologies. Although the cells showed similar morphology, being elongated and attached to the surface through the elongations, the cell density was lower on some of the surfaces which could hamper the cell-cell interaction that is required to promote cell proliferation and the growth of a new tissue in an *in vivo* model (Figure 7).

**Figure 8:**
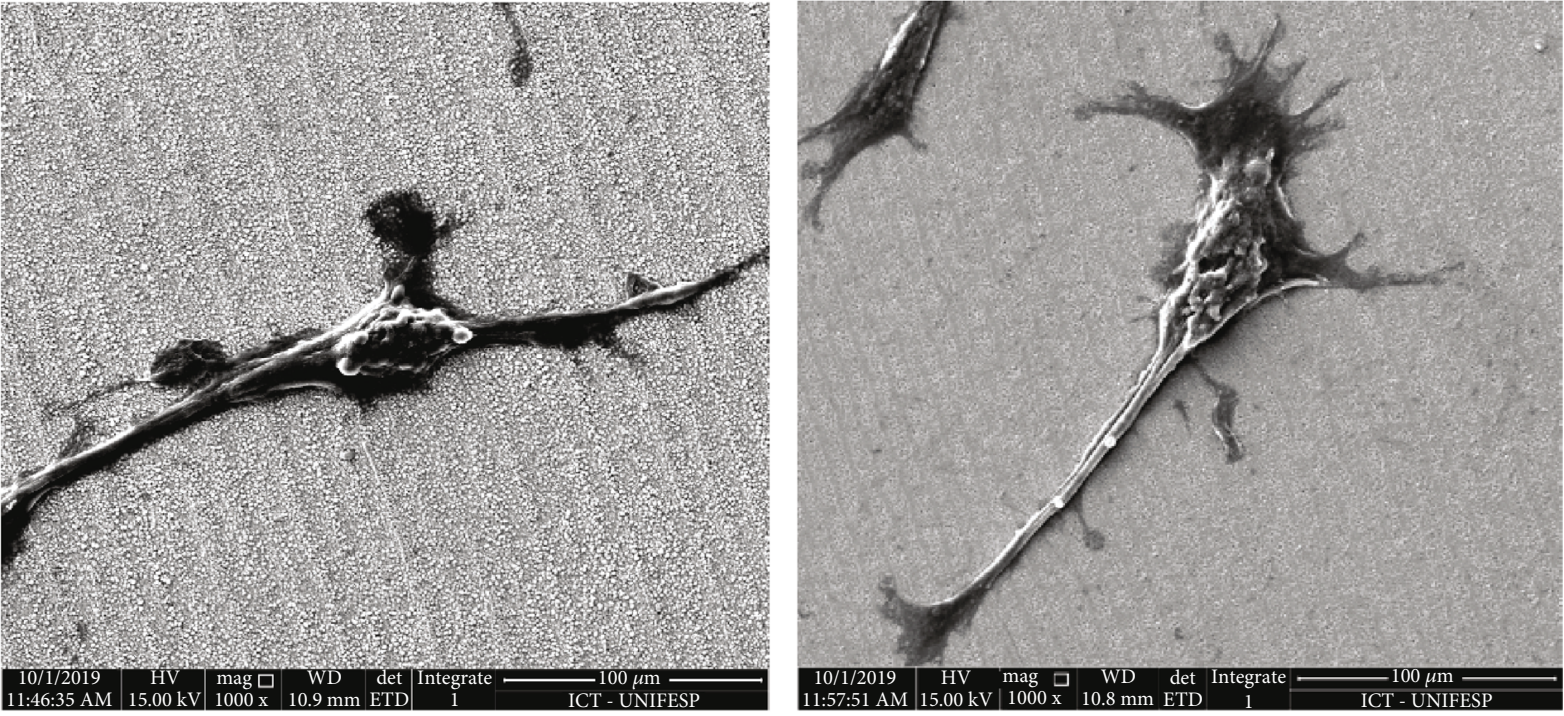
SEM of elongated shape of the adhered cell in quadrants 3 and 4.

Even if the cell density was lower, the cell morphology suggested that these cells were prone to proliferate on the surface. Probably, by using higher cell concentration in the cell, seeding step and longer incubation time would be enough to observe higher cell coverage on these surfaces. Even so, these preliminary assays were valuable to show that these surfaces were suitable for the cell adhesion. The elongated shape of the fibroblasts was observed on several LIPSS formed (Figure 8).

**Figure 9:**
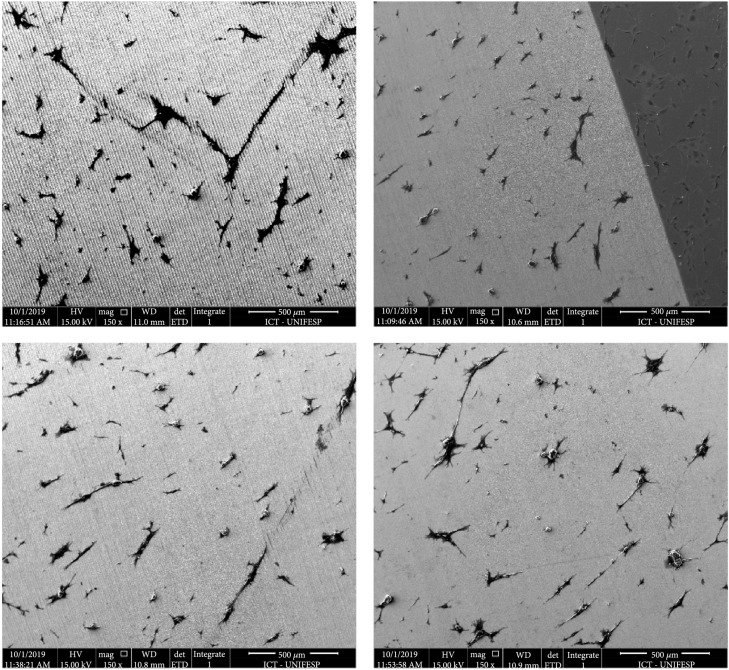
SEM of general cell dispersion in quadrants 1 and 2 (upper row) and 3 and 4 (below).

The number of elongated cells on the different topologies was quantified in the SEM images (Figure 9). Since this morphology is suitable for the cell-cell contact and to induce cell proliferation, it was used as a tool to evaluate which topology would be the most appropriated in order to induce the development of a new tissue.

It is possible to compare the four quadrants presented in Figure 9 and conclude that they have a similar cell dispersion between them. The number of cells adhered can be considered equal by the whole surface. But the elongated shape, found in the four quadrants, is more numerically represented in quadrants three and four. They also have an oriented growth that means that in general the cells were grown in the same direction, whereas in quadrants one and two there were adhered cells with elongated shape, but fewer elongated cells and with different orientations.

## 5. Discussion and Conclusions

The main goal of this work is to create a surface that can promote cell adhesion and the growth of endothelial cells using a laser structuring method that is very clean and highly controllable with structure sizes in the micron- and submicron range.

Therefore, authors expect that the VADs can present better results with less thrombus formation and hemolysis rates. Instead of a passive surface obtained by polished Titanium, a bio-inert material, like so many successful VADs in the market, our research concerns a new type of biomaterial that could show enhanced bioactivity. The present work consists in the first step towards these aims, and therefore, it shows preliminary biocompatibility assay with MEF cells.

Some approaches that have already been investigated include coating titanium with polymer, which faced the limitation of coating detachment, or other surface modifications to obtain hydrophobic surface *per se*. Differently, the present work is focused on the creation of the surface topology and evaluate the ability of cells to adhere to them. As preliminary results, the interaction with fibroblasts was evaluated and they motivated the further assays that will be performed with blood cells.

Differently, the present work is focused on the progressive study of the surface topology influence on the cell interaction. As preliminary results, the interaction with fibroblasts was evaluated and they motivated the further assays that will be performed with blood cells.

As can be evidenced, the LIPSS were created on the titanium surface. The SEM images show that the cells were properly adhered on the surface and with an elongated shape characteristic of fibroblasts. The cell proliferation will be further evaluated by the in vitro assays for longer periods. Nevertheless, these preliminary results pointed that the proposed LIPSS topology would be the most appropriated surface to induce the growth of cells and the development of new tissue.

## Data Availability

The SEM data used to support the findings of this study have been deposited in the Research Gate repository (doi:10.13140/RG.2.2.19689.98402).
